# Markedly elevated bone mineral density in genetically elusive familial hypocalciuric hypercalcemia: A case report with targeted genetic analysis of eight candidate genes

**DOI:** 10.1016/j.bonr.2026.101914

**Published:** 2026-03-31

**Authors:** Shuichi Yatsuga, Yutaka Kozakai

**Affiliations:** a Department of Medical Genetics, Hakodate Goryoukaku Hospital, 38-3 Goryokaku-cho, Hakodate-City, 040-8611, Hokkaido, Japan; b Department of Pediatrics, Fukuoka University, 7-45-1 Nanakuma, Jonan-ku, Fukuoka-City, 810-0180, Fukuokao, Japan; c Department of Orthopedics, Hakodate Goryoukaku Hospital, 38-3 Goryokaku-cho, Hakodate-City, 040-8611, Hokkaido, Japan

**Keywords:** Familial hypocalciuric hypercalcemia, Bone mineral density, *CASR*, Parathyroid hormone, Genetic testing

## Abstract

We report an 88-year-old Japanese woman with markedly elevated bone mineral density (lumbar spine 212% of young adult mean, Z–score + 10.0 SD) diagnosed with familial hypocalciuric hypercalcemia based on a urinary calcium-to-creatinine ratio of 0.06. Tergeted genetic testing of eight genes revealed no pathogenic variants; a *CASR* variant (c.1733-9A>G) was a common Japanese polymorphism (frequency 1/808). This genetically elusive case highlights the extreme skeletal phenotype in FHH and the essential role of population-specific databases in variant interpretation. ©The Authors. All rights reserved.

## Highlights

• An 88-year-old FHH patient exhibited lumbar spine BMD of 212% (Z-score + 10.0 SD).
• Targeted genetic analysis of eight genes identified no pathogenic variants.
• The *CASR* c.1733-9A>G variant was confirmed benign in Japanese databases.
• Extreme BMD likely reflects cumulative PTH anabolic effects over decades.
• Population-specific databases are crucial for accurate FHH variant interpretation.

## 1 Introduction

Familial hypocalciuric hypercalcemia (FHH) is a benign autosomal dominant disorder caused by heterozygous inactivating mutations in genes encoding the calcium-sensing receptor (*CASR*, FHH1), Gα11 subunit (*GNA11*, FHH2), and adaptor protein complex 2 sigma subunit (*AP2S1*, FHH3) (Pollak et al., 1993; Nesbit et al., 2013a; Nesbit et al., 2013b). These mutations result in decreased sensitivity to extracellular calcium, leading to inappropriate parathyroid hormone (PTH) secretion and reduced renal calcium excretion (Hannan et al., 2018).

The skeletal phenotype associated with Familial Hypocalciuric Hypercalcemia (FHH) has been extensively studied. The markedly elevated BMD observed in this patient is closely associated with the lifelong mild elevation on PTH levels characteristic of FHH. The anabolic effects of PTH on bone are well-documented (Neer et al., 2001; Rubin and Bilezikian, 2005), and chronic mild PTH elevation in FHH may exert cumulative effects over extended periods. However, we acknowledge that this association dose not establish direct causation. Multiple factors likely contribute to the skeletal phenotype, including: (1) calcium-sensing receptor dysfunction in bone cells, (2) altered calcium homeostasis, (3) genetic background factors, and (4) potential unknown effects of the underlying genetic variant.

Despite the identification of three FHH genes, approximately 10–30% of clinically diagnosed FHH patients lack pathogenic variants in known genes, suggesting additional genetic heterogeneity (Asla et al., 2024; Hinnie et al., 2001; Thakker, 2016). These “genetically elusive” cases pose diagnostic challenges and offer opportunities for novel gene discovery.

We report a case of genetically elusive FHH with extraordinarily elevated BMD (>200% of age-matched mean), representing an extreme example of the skeletal phenotype in this condition. This case underwent targeted genetic testing of eight genes and highlights the critical importance of population-specific variant databases in genetic interpretation.

## 2 Case

Comprehensive family history could not be conclusively determined due to the refusal of detailed genetic testing by family members. The patient has two adult offspring who report no history of significant illness or symptoms indicative of hypercalcemia, such as nephrolithiasis, bone pain, or pathological fractures. Neither offspring has undergone biochemical screening for disorders related to calcium metabolism.

Genetic variants were categorized in accordance with the guidelines established by the American College of Medical Genetics and Genomics (ACMG) and the Association for Molecular Pathology (AMP) (Richards et al., 2015).

An 88-year-old Japanese woman presented to orthopedics with left groin pain and was diagnosed with pubic insufficiency fracture. Incidental laboratory findings revealed hypercalcemia (ionized calcium 3.07 mmol/L, reference 2.41–2.72) and elevated intact PTH (98 pg/mL, reference <65), prompting referral to medical genetics. She had been asymptomatic throughout her life with no history of nephrolithiasis, bone pain, or previous pathological fractures. Medical history included hypertension. Family history could not be definitively established as family screening was declined. Physical examination was unremarkable except for tenderness over the left pubic bone.

Serial biochemical studies confirmed persistent hypercalcemia and elevated PTH (Table 1). Crucially, the urinary calcium-to-creatinine ratio was 0.06, which was markedly below the threshold for PHPT (>0.2) and highly suggestive of FHH (Christensen et al., 2011; Eastell et al., 2014).

**Table 1:** Serial laboratory findings.

Parameter	Values obtained one month after pelvic fracture	Reference Range
Calcium metabolism		
Ionized calcium (mmol/L)	3.07	2.41–2.72
Total calcium (mg/dL)	9.6	8.8–10.2
Phosphorus (mg/dL)	4.3	2.5–4.5
Intact PTH (pg/mL)	98	<65

Urinary calcium		
Urine Ca/Cr ratio	0.06	>0.2

Bone turnover markers		
Total ALP (IU/L)	207	38–113
BAP (μg/L)	41.5	3.8–22.6
TRACP-5b (mU/dL)	280	120–420

Vitamin D		
25(OH)D (ng/mL)	26	30–100

Renal function		
Creatinine (mg/dL)	0.73	0.4–0.8
eGFR (mL/min/1.73m^2^)	56	>60

ALP, alkaline phosphatase; BAP, bone-specific alkaline phosphatase; TRACP-5b, tartrate-resistant acid phosphatase 5b; 25(OH)D, 25-hydroxyvitamin D; PTH, parathyroid hormone; eGFR, estimated glomerular filtration rate.

Bone turnover markers were elevated: alkaline phosphatase (ALP) 207 IU/L (reference 38–113), bone-specific ALP (BAP) 41.5 μg/L (reference 3.8–22.6), and tartrate-resistant acid phosphatase 5b (TRACP-5b) 280 mU/dL (reference 120–420), suggesting increased bone remodeling. The serum 25-hydroxyvitamin D level was 26 ng/mL (insufficient range).

**Fig. 1:**
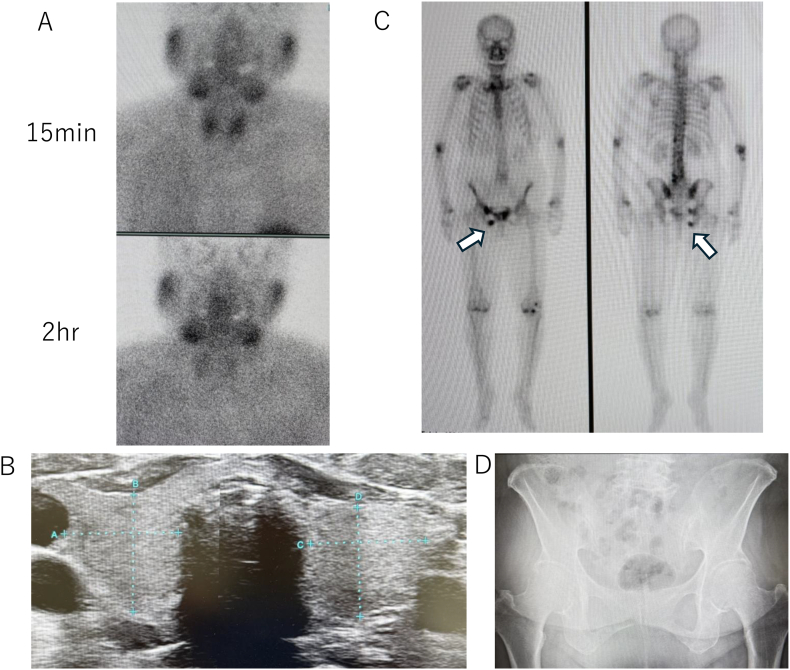
Imaging studies. (D) Pelvic radiography demonstrating sclerotic changes at the pubic fracture site (arrow). Initially, these findings suggested the possibility of Paget's disease; however, they were ultimately determined to be indicative of fracture healing.

^99^mTc-MIBI parathyroid scintigraphy revealed no evidence of parathyroid adenoma (Fig. 1A). Thyroid ultrasonography revealed no discrete parathyroid enlargement (Fig. 1B). Approximately one month subsequent to the pubic fracture, a ^99^mTc-methylene diphosphonate (MDP) bone scintigraphy was conducted, which demonstrated a focal increase in uptake in the left pubic bone, while no abnormal uptake was observed in the lumber spine or femoral regions (Fig. 1C). Pelvic radiography demonstrated focal sclerotic changes at the fracture site (Fig. 1D). The initial clinical suspicion of monostotic Paget's disease was prompted by the combination of focal scintigraphic uptake, elevated ALP levels (207 IU/L), increased TRACP-5b (280 mU/dL), and sclerotic radiographic changes. However, the subsequent spontaneous normalization of ALP levels, in the absence of treatment specific to Paget's disease, suggesting that these findings were indicative of the normal fracture healing process rather an underlying case of Paget's disease.

**Fig. 2:**
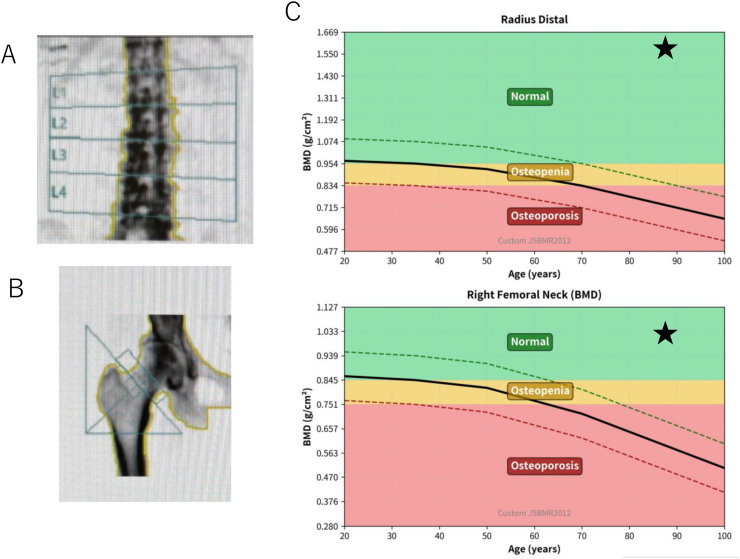
Bone mineral density assessment. (A) Lumbar spine DXA demonstrating a BMD of 1.596 g/cm^2^ (212% of the young adult mean, *Z*-score + 10.0 SD). (B) Femoral neck DXA demonstrating a BMD of 0.977 g/cm^2^ (172% of age-matched mean, Z-score + 6.0 SD). (C) Graphical representation comparing the patient's BMD to age-matched reference ranges, illustrating extreme elevation at both skeletal sites.

Dual-energy X-ray absorptiometry (DXA) revealed remarkably elevated BMD (Fig. 2): lumbar spine (L2-L4) 1.596 g/cm^2^ (212% of the young adult mean, *Z*-score + 10.0 SD), and femoral neck 0.977 g/cm^2^ (172% of the age-matched mean, 104% of the young adult mean, Z-score + 6.0 SD). These values represent some of the highest reported BMD measurements in FHH, illustrating the extreme skeletal phenotype possible with a lifelong mild PTH elevation. This case illustrates the paradox of fracture occurrence despite an extremely high BMD indicated by a Z-score + 10.0 SD. In such scenarios, it is crucial to consider conditions in the differential diagnosis where fracture risk is elevated despite high BMD, particularly Paget's disease. In this instance, monostotic Paget's disease was included in the differential diagnosis due to the presence of high BMD, fracture, bone pain, and focal abnormalities observed in the bone scan.

Targeted next-generation sequencing with Sanger confirmation was performed for eight genes: *CASR*, *GNA11*, *AP2S1* (FHH-associated), *MEN1*, *RET*, *CDKN1B*, *CDC73*, and *GCM2* (other familial hypercalcemia-associated genes). A heterozygous *CASR* variant (NM_000388.4:c.1733-9 A > G) was identified. However, population database analysis revealed a high frequency in Japanese cohorts: ToMMo 61KJPN 0.001238 (1/808), gnomAD East Asian 0.001181(1/847), and gnomAD Global 0.000440 (1/2273). Two homozygous individuals were identified in the gnomAD exome data. Since homozygous *CASR* inactivation causes life-threatening severe hyperparathyroidism in neonates [14], the presence of asymptomatic adult homozygotes definitively establishes this variant as benign. In silico splicing prediction tools (SpliceAI score 0.0) confirmed that there was no impact on splicing. No other pathogenic or likely pathogenic variants were identified (Table 2). (See Table 3.)

**Table 2:** Targeted genetic testing results.

Gene	Disease association	Inheritance	Testing result	Classification
*CASR*	FHH type 1	AD	c.1733-9 A > G (het)	Benign variant
*GNA11*	FHH type 2	AD	Negative	No pathogenic variant
*AP2S1*	FHH type 3	AD	Negative	No pathogenic variant
*MEN1*	MEN1 syndrome	AD	Negative	No pathogenic variant
*RET*	MEN2 syndrome	AD	Negative	No pathogenic variant
*CDKN1B*	MEN4 syndrome	AD	Negative	No pathogenic variant
*CDC73*	HPT-JT syndrome	AD	Negative	No pathogenic variant
*GCM2*	Hypoparathyroidism	AR	Negative	No pathogenic variant

FHH, familial hypocalciuric hypercalcemia; MEN, multiple endocrine neoplasia; HPT-JT, hyperparathyroidism-jaw tumor syndrome; AD, autosomal dominant; AR, autosomal recessive; het, heterozygous.

**Table 3:** *CASR* c.1733-9A>G population frequency data.

Population	Allele frequency	Allele count	Homozygotes	Database
Japanese (ToMMo 61 K)	0.001238 (1/808)	152/122,742	0	jMorp
East Asian (gnomAD)	0.001181 (1/847)	50/44,874	–	gnomAD v4
Global (gnomAD exome)	0.000440 (1/2273)	679/1,460,364	2	gnomAD v4

No functional studies concerning the c.1733-9 A > G variant have been documented in the literature. In silico analyses employing multiple splice prediction algorithms, including SpliceAI, and MaxEntScan, suggesting no anticipated impact on mRNA splicing. The variant's deep intronic position, located 9 base pairs from the exon boundary, situates it outside the canonical splice consensus sequence.

Based on the pathognomonic urinary calcium-to-creatinine ratio of 0.06, lifelong asymptomatic course, negative parathyroid imaging, markedly elevated BMD, and absence of pathogenic variants in eight known genes, a clinical diagnosis of genetically elusive familial hypocalciuric hypercalcemia was established.

Parathyroidectomy was therefore contraindicated. The patient continues to undergo annual surveillance with stable calcium levels and preserved renal function. The patient remained clinically well, with no complications from hypercalcemia, after 12 months of follow-up.

The skeletal phenotype associated with Familial Hypocalciuric Hypercalcemia (FHH) has been extensively documented in the literature, with bone mineral density (BMD) generally reported as normal to modestly elevated (Yamamoto et al., 1988; Christensen et al., 2009). Yamamoto et al. (1988) were the first to report higher BMD in FHH compared to Primary Hyperparathyroidism (PHPT). Christensen et al. (2009) noted that the mean lumbar spine BMD in FHH was approximately 110–120% of age-matched controls, significantly surpassing that of PHPT patients, which ranged from 90 to 95%. Individual case reports have recorded BMD values ranging from 100% to approximately 180% of age-matched means. Our patient's BMD, measured at 212% (lumbar spine) and 172% (femoral neck) of age-matched means, represents one of the highest values documented in the FHH literature, exemplifying the extreme end of the phenotypic spectrum. This remarkable elevation illustrates the cumulative anabolic effects of 88 years of sustained mild parathyroid hormone (PTH) elevation. The divergent skeletal phenotypes of FHH and PHPT are attributed to fundamental differences in calcium balance. In PHPT, renal calcium losses are elevated and typically exceed intestinal absorption, resulting in a net negative calcium balance that contributes to bone resorption and osteopenia (Silverberg et al., 1999). Conversely, FHH is characterized by renal calcium retention due to decreased calcium-sensing receptor function in the kidney. This preserved or positive calcium balance, coupled with sustained mild PTH elevation, creates conditions conducive to net bone formation. The extraordinarily high BMD in our patient reflects 88 years of this favorable calcium balance, demonstrating the cumulative impact of altered calcium homeostasis on skeletal health.

## 3 Discussion

Although three genes associated with familial hypocalciuric hypercalcemia (FHH)—*CASR*, *GNA11*, and *AP2S1*—have been identified, approximately 10–30% of patients clinically diagnosed with FHH do not exhibit pathogenic variants in these known genes (Asla et al., 2024; Hinnie et al., 2001; Thakker, 2016). These ‘genetically elusive’ cases indicate additional genetic heterogeneity and present opportunities for the discovery of novel genes.

This case demonstrates an extraordinarily elevated BMD (212% of young adult mean in lumbar spine), representing one of the highest reported values in FHH literature. This skeletal phenotype contrasts sharply with that of PHPT, in which continuous PTH excess typically causes cortical bone loss and osteopenia (Silverberg et al., 1999; Khosla et al., 1999). The bone-protective effect in FHH can be explained by several mechanisms. While continuous PTH excess in PHPT causes net bone resorption, the mild, lifelong PTH elevation in FHH (median PTH ∼80–100 pg/mL) appears to exert predominantly anabolic effects (Yamamoto et al., 1988; Carling et al., 2000), mirroring the mechanism of intermittent PTH administration (Neer et al., 2001; Rubin and Bilezikian, 2005). Our patient's 88-year exposure to mild PTH elevation allowed cumulative anabolic effects on bone mass.

The marked difference in skeletal phenotype between FHH and PHPT has important diagnostic implications. Studies have shown that patients with FHH have significantly higher BMD than those with PHPT at all skeletal sites (Yamamoto et al., 1988; Carling et al., 2000; Christensen et al., 2009). In our case, the extraordinarily high BMD (>200% of age-matched mean) strongly suggested FHH rather than PHPT, even before genetic testing. We propose that BMD assessment should be incorporated into diagnostic algorithms for differentiating FHH from PHPT, combined with the urinary calcium-to-creatinine ratio for optimal discrimination (Christensen et al., 2011; Eastell et al., 2014).

Despite testing of eight genes, no pathogenic variant was identified, representing approximately 10–30% of clinically diagnosed FHH cases (Asla et al., 2024; Hinnie et al., 2001; Thakker, 2016). Candidate mechanisms include novel FHH genes in calcium-sensing or PTH secretion pathways (Gorvin, 2018), regulatory variants in promoter or enhancer regions (Hannan et al., 2015a, Hannan et al., 2015b), structural variants missed by standard sequencing (Vargas-Poussou et al., 2002), somatic mosaicism (Lemos and Thakker, 2008), and oligogenic inheritance (Hendy et al., 2013).

This case underscores the significance of longitudinal observations in distinguishing Paget's disease from fracture healing. Both conditions may exhibit similar clinical manifestations, including focal bone scan abnormalities, elevated bone turnover markers, and sclerotic bone changes. At one month post-fracture, it remains challenging to achieve complete differentiation based solely on these findings. As demonstrated by Frost (1989), the 4-week post-fracture period constitutes the most active phase of bone remodeling, during which an elevation of bone turnover markers is physiological. However, in clinical practice, distinguishing this physiological change from Paget's disease when focal imaging abnormalities are present is not straightforward. This case underscores the importance of assessing changes in findings over time. In this instance, both Paget's disease and fracture healing were included in the initial differential diagnosis, and the patient was carefully monitored without treatment, such as bisphosphonates. This approach confirmed the spontaneous normalization of bone turnover markers, leading to a definitive diagnosis of fracture healing. This diagnostic process exemplifies the achievement of an accurate diagnosis while avoiding overtreatment. Paget's disease is characterized by markedly elevated BMD with increased fracture risk due to impaired bone quality, often accompanied by bone pain, elevated bone turnover markers, and focal bone scan abnormalities. These features matched the clinical presentation of this case (high BMD + fracture + bone pain + elevated markers). Therefore, including Paget's disease in the initial differential diagnosis was appropriate clinical practice. Despite extremely high BMD (lumbar spine *Z*-score + 10.0 SD, femoral neck Z-score + 6.0 SD), this patient sustained a pubic fracture from minor trauma. This provides important clinical insights regarding determinants of bone strength.

While bone mineral density (BMD) is strongly correlated with fracture risk at the population level (Schuit et al., 2004; Stone et al., 2003), it does not perfectly predict fracture occurrence in individual patients. This limitation arises because bone strength is determined not only by BMD (bone quantity) but also by bone quality. Bone quality encompasses factors such as microarchitecture (trabecular structure, cortical porosity), collagen organization (cross-linking, orientation), the degree and distribution of mineralization, the accumulation of microdamage, and the rate of bone turnover. The calcium-sensing receptor is expressed in osteoblasts and osteocytes (Gorvin, 2018; Hannan et al., 2018), and its dysfunction may influence bone material properties beyond its effects on BMD.

At the age of 88, several factors contribute to an increased risk of fractures, including qualitative changes in collagen cross-linking due to the accumulation of advanced glycation end-products, the accumulation of microdamage, an elevated risk of falls, and weakened protective mechanisms resulting from decreased muscle strength. In familial hypocalciuric hypercalcemia (FHH), long-term abnormalities in calcium metabolism may lead to brittleness due to excessive mineralization (hypermineralization), where a high quantity does not necessarily ensure optimal quality. Alterations in bone microarchitecture, impairment of the normal bone remodeling cycle, and changes in collagen-mineral interactions may also play significant roles. The pubic bone, characterized by thin cortical bone, differs in structure and composition from sites typically measured by DXA, such as the lumbar spine and femoral neck, indicating site-specific vulnerability. Additionally, the direction and magnitude of impact force during falls, reduced cushioning by soft tissue, and concentrated loading all contribute to fracture risk in elderly individuals.

Instances of fractures occurring despite elevated bone mineral density (BMD) have been documented in conditions such as Paget's disease, where increased fracture risk is attributed to microarchitectural abnormalities; fluorosis, characterized by bone fragility despite augmented bone mass due to hypermineralization; and osteopetrosis, which involves high bone mass but diminished bone quality. In such cases, excessive mineralization may render the bone excessively hard and brittle, similar to the phenomena observed in fluorosis. Abnormal bone remodeling resulting from CASR dysfunction may contribute to the accumulation of microdamage over an 88-year period. This case illustrates that even extreme BMD values do not confer complete protection against fractures, particularly in the context of metabolic bone disorders. It underscores the necessity of evaluating bone quality in addition to bone quantity when assessing bone strength and fracture risk.

The mechanisms underlying the patient's exceptionally high BMD, as indicated by a lumbar spine *Z*-score of +10.0 SD and a femoral neck Z-score of +6.0 SD, are likely attributable to the synergistic effects of multiple factors. First, the cumulative promotion of bone formation due to sustained mild elevation of PTH levels (64.6 pg/mL) over a period of 88 years is a probable contributing factor. While intermittent PTH therapy, such as teriparatide, has been shown to increase lumbar spine BMD by 9–13% over 18–24 months (Neer et al., 2001), the prolonged duration of 88 years in this case may have resulted in more pronounced cumulative effects. It is important to note that teriparatide involves intermittent administration, whereas this case involves sustained elevation, potentially altering the balance between anabolic and catabolic effects. The extremely high BMD phenotype suggests that PTH elevation in FHH primarily exerts an anabolic effect. Second, the CASR is expressed in osteoblasts and osteocytes (Gorvin, 2018; Hannan et al., 2018), and its dysfunction can lead to alterations in intracellular calcium signaling within bone cells. This may affect the balance between bone formation and resorption, thereby contributing to increased bone mass. Additionally, CASR dysfunction may influence the promotion of bone matrix mineralization. Third, polymorphisms in other genes related to bone metabolism, such as *LRP5*, *SOST*, *VDR*, and *COL1A1*, may modify the FHH phenotype, and epigenetic modifications and unidentified genetic modifiers may also play a role. Over the course of 88 years, factors such as nutritional status, physical activity levels, and hormonal environment (including post-menopausal estrogen decline) may have interacted with PTH elevation and CASR dysfunction to influence the skeletal phenotype.

In FHH, BMD is generally normal to mildly elevated (Marx et al., 1981; Khosla et al., 1999). The extreme phenotype observed in this case is likely attributable to a unique combination of these factors. This case serves as a rare natural experiment, illustrating the skeletal effects of sustained mild PTH elevation over an extended period.

The pathogenic significance of the *CAS*R c.1733-9 A > G variant necessitates careful evaluation based on current database information. Although this variant is located at position −9 and could theoretically influence splicing, multiple prediction tools, including SpliceAI, yield scores near 0, indicating no impact on splicing. Furthermore, recent clinical laboratory assessments in ClinVar (Labcorp, Mayo Clinic, Copenhagen University Hospital) consistently classify it as benign/likely benign according to ACMG criteria (BP4: no impact by computational evidence, BP7: silent variant). The population frequency (MAF 0.08%) also exceeds that typically associated with pathogenic variants. Based on this evidence, this variant is classified as benign/likely benign, and its direct contribution to the FHH phenotype in this case appears limited. However, the potential involvement of unidentified genetic factors contributing to the phenotype, particularly exceptional BMD, cannot be excluded. Further functional studies, including RNA analysis, would be valuable to definitively assess the impact of this variant on CASR function.

Patients diagnosed with FHH necessitate ongoing surveillance despite the condition's benign nature. Essential monitoring parameters include: (1) Annual evaluation of serum calcium and PTH levels to identify any progression, which is rare in FHH; (2) Assessment of renal function due to chronic mild hypercalcemia, although the risk of nephrolithiasis remains low (Marx et al., 1981); (3) Monitoring of BMD to evaluate age-related changes, as the protective effect of PTH may be offset by estrogen deficiency in postmenopausal women; (4) Avoidance of unnecessary parathyroid surgery, which poses risks of permanent hypoparathyroidism and persistent hypercalcemia (Attie et al., 1983). Genetic counseling for offspring is advised, highlighting the benign prognosis and the autosomal dominant inheritance pattern.

The *CASR* c.1733-9 A > G variant illustrates the essential role of population-specific databases in genetic interpretation (Manrai et al., 2016; Tadaka et al., 2018). Without population data, this splice region variant might have been considered pathogenic. However, Japanese population databases definitively established its benign nature through high allele frequency (1/808) and presence of asymptomatic adult homozygotes. This underscores the critical need for the expansion of non-European population databases, mandatory use of ancestry-matched controls, and recognition that variant pathogenicity is population-specific (Manrai et al., 2016; Tadaka et al., 2018).

Even without genetic confirmation, the clinical diagnosis was sufficiently certain to contraindicate parathyroidectomy. Historical reports have documented severe complications, including persistent hypercalcemia, severe hypocalcemia, and permanent hypoparathyroidism (Attie et al., 1983; Marx et al., 1981). FHH has an excellent prognosis, with no progression of hypercalcemia, low risk of nephrolithiasis, no increased fracture risk, and normal life expectancy (Hannan et al., 2018; Forde et al., 2014).

This case report has several limitations: family screening could not be performed; whole-genome sequencing was not performed; RNA analysis was not available; historical calcium values were unavailable. Despite these limitations, the clinical diagnosis remains highly certain based on the pathognomonic urinary calcium-to-creatinine ratio of 0.06.

This case illustrates several important principles: (1) Extreme skeletal phenotype in FHH: BMD exceeding 200% of age-matched mean (*Z*-score + 10.0 SD at lumbar spine, +6.0 SD at femoral neck) reflects the potent, lifelong anabolic effects of sustained mild PTH elevation, contrasting sharply with osteopenia in PHPT. (2) Diagnostic value of BMD: Skeletal assessment should be incorporated into diagnostic algorithms for differentiating FHH from PHPT. (3) Genetically elusive FHH is common: Despite targeted testing of known genes, ∼10–30% of cases remain unexplained. (4) Population databases are essential: The CASR variant was definitively established as benign only through Japanese population data. (5) Clinical diagnosis supersedes genetics: The pathognomonic urinary calcium-to-creatinine ratio established the diagnosis with certainty. This patient's DNA represents a valuable resource for future genomic studies aimed at identifying novel FHH genes.

The DNA of this patient constitutes a significant resource for prospective genomic research. Whole-genome sequencing conducted through collaborative research networks may facilitate the identification of novel FHH genes and enhance our comprehension of genetic heterogeneity in calcium-sensing disorders. Although targeted analysis of known candidate genes did not uncover the causative variant, the preservation of patient DNA allows for future comprehensive genomic investigations when resources and infrastructure become available.

## Ethical form

The patient was informed that the case data would be submitted for publication, and she provided written consent.

## CRediT authorship contribution statement

**Shuichi Yatsuga:** Writing – review & editing, Writing – original draft, Validation, Supervision, Investigation, Data curation, Conceptualization. **Yutaka Kozakai:** Writing – review & editing, Supervision, Conceptualization.

## Declaration of Generative AI and AI-assisted technologies in the writing process

During the preparation of this work, the author used Claude and Paperpal to refine the English. After using this tool/service, the authors reviewed and edited the content as needed and took full responsibility for the content of the published article.

## Declaration of competing interest

The authors declare that they have no competing financial interests or personal relationships that could have influenced the work reported in this paper.

## Data Availability

Data will be made available on request.

## References

[bb0005] Asla Q., Sardà H., Seguí N., Martínez de Pinillos G., Mazarico-Altisent I., Capel I., Aulinas A. (2024). Clinical and outcome comparison of genetically positive vs. negative patients in a large cohort of suspected familial hypocalciuric hypercalcemia. Endocrine.

[bb0010] Attie M.F., Gill J.R., Stock J.L., Spiegel A.M., Downs R.W., Levine M.A., Marx S.J. (1983). Urinary calcium excretion in familial hypocalciuric hypercalcemia. Persistence of relative hypocalciuria after induction of hypoparathyroidism. J. Clin. Invest..

[bb0015] Carling T., Szabo E., Bai M., Friedman E., Grodski S., Kohlin M., Enberg U., Hessman O., Teh B.T., Farnebo L.O., Rastad J. (2000). Familial hypercalcemia and hypercalciuria caused by a novel mutation in the cytoplasmic tail of the calcium receptor. J. Clin. Endocrinol. Metab..

[bb0020] Christensen S.E., Nissen P.H., Vestergaard P., Heickendorff L., Rejnmark L., Brixen K., Mosekilde L. (2009). Skeletal consequences of familial hypocalciuric hypercalcaemia vs. primary hyperparathyroidism. Clin. Endocrinol..

[bb0025] Christensen S.E., Nissen P.H., Vestergaard P., Mosekilde L. (2011). Familial hypocalciuric hypercalcaemia: a review. Curr. Opin. Endocrinol. Diabetes Obes..

[bb0030] Eastell R., Brandi M.L., Costa A.G., D’Amour P., Shoback D.M., Thakker R.V. (2014). Diagnosis of asymptomatic primary hyperparathyroidism: proceedings of the fourth international workshop. J. Clin. Endocrinol. Metab..

[bb0035] Forde H.E., Hill A.D., Smith D. (2014). Parathyroid adenoma in a patient with familial hypocalciuric hypercalcaemia. BMJ Case Rep..

[bb0040] Frost H.M. (1989). The biology of fracture healing. An overview for clinicians. Part I. Clin. Orthop. Relat. Res..

[bb0045] Gorvin C.M. (2018). Insights into calcium-sensing receptor trafficking and biased signalling by studies of calcium homeostasis. J. Mol. Endocrinol..

[bb0050] Hannan F.M., Howles S.A., Rogers A., Cranston T., Gorvin C.M., Babinsky V.N., Reed A.A., Thakker C.E., Bockenhauer D., Brown R.S., Connell J.M., Cook J., Darzy K., Ehtisham S., Winer K., Kohlhase J., Hisado-Oliva A., Giner T., Alonso M.P., Denes F.T., Beltrán C., Plöckinger U., Jüppner H., Thakker R.V. (2015). Adaptor protein-2 sigma subunit mutations causing familial hypocalciuric hypercalcaemia type 3 (FHH3) demonstrate genotype-phenotype correlations, codon bias and dominant-negative effects. Hum. Mol. Genet..

[bb0055] Hannan F.M., Walls G.V., Babinsky V.N., Nesbit M.A., Kallay E., Hough T.A., Fraser W.D., Cox R.D., Hu J., Spiegel A.M., Thakker R.V. (2015). The calcilytic agent NPS 2143 rectifies hypocalcemia in a mouse model with an activating calcium-sensing receptor (CaSR) mutation: relevance to autosomal dominant hypocalcemia type 1 (ADH1). Endocrinology.

[bb0060] Hannan F.M., Kallay E., Chang W., Brandi M.L., Thakker R.V. (2018). The calcium-sensing receptor in physiology and in calcitropic and noncalcitropic diseases. Nat. Rev. Endocrinol..

[bb0065] Hendy G.N., Canaff L., Cole D.E. (2013). The CASR gene: alternative splicing and transcriptional control, and calcium-sensing receptor (CaSR) protein: structure and ligand binding sites. Best Pract. Res. Clin. Endocrinol. Metab..

[bb0070] Hinnie J., Bell E., McKillop E., Gallacher S. (2001). The prevalence of familial hypocalciuric hypercalcemia. Calcif. Tissue Int..

[bb0075] Khosla S., Melton L.J., Wermers R.A., Crowson C.S., O'Fallon W.M., Riggs B.L. (1999). Primary hyperparathyroidism and the risk of fracture: a population-based study. J. Bone Miner. Res..

[bb0080] Lemos M.C., Thakker R.V. (2008). HRPT2 mutations are associated with malignancy in sporadic parathyroid tumours. Clin. Endocrinol..

[bb0085] Manrai A.K., Funke B.H., Rehm H.L., Olesen M.S., Maron B.A., Szolovits P., Margulies D.M., Loscalzo J., Kohane I.S. (2016). Genetic misdiagnoses and the potential for health disparities. N. Engl. J. Med..

[bb0095] Marx S.J., Attie M.F., Levine M.A., Spiegel A.M., Downs R.W., Lasker R.D. (1981). The hypocalciuric or benign variant of familial hypercalcemia: clinical and biochemical features in fifteen kindreds. Medicine (Baltimore).

[bb0105] Neer R.M., Arnaud C.D., Zanchetta J.R., Prince R., Gaich G.A., Reginster J.Y., Hodsman A.B., Eriksen E.F., Ish-Shalom S., Genant H.K., Wang O., Mitlak B.H. (2001). Effect of parathyroid hormone (1-34) on fractures and bone mineral density in postmenopausal women with osteoporosis. N. Engl. J. Med..

[bb0110] Nesbit M.A., Hannan F.M., Howles S.A., Babinsky V.N., Head R.A., Cranston T., Rust N., Hobbs M.R., Heath H., Thakker R.V. (2013). Mutations affecting G-protein subunit α11 in hypercalcemia and hypocalcemia. N. Engl. J. Med..

[bb0115] Nesbit M.A., Hannan F.M., Howles S.A., Reed A.A., Cranston T., Thakker C.E., Gregory L., Rimmer A.J., Rust N., Graham U., Morrison P.J., Hunter S.J., Whyte M.P., McVean G., Buck D., Thakker R.V. (2013). Mutations in AP2S1 cause familial hypocalciuric hypercalcemia type 3. Nat. Genet..

[bb0120] Pollak M.R., Brown E.M., Chou Y.H., Hebert S.C., Marx S.J., Steinmann B., Levi T., Seidman C.E., Seidman J.G. (1993). Mutations in the human Ca(2+)-sensing receptor gene cause familial hypocalciuric hypercalcemia and neonatal severe hyperparathyroidism. Cell.

[bb0130] Richards S., Aziz N., Bale S., Bick D., Das S., Gastier-Foster J., Grody W.W., Hegde M., Lyon E., Spector E., Voelkerding K., Rehm H.L. (2015). Standards and guidelines for the interpretation of sequence variants: a joint consensus recommendation of the American College of Medical Genetics and Genomics and the Association for Molecular Pathology. Genet. Med..

[bb0135] Rubin M.R., Bilezikian J.P. (2005). Parathyroid hormone as an anabolic skeletal therapy. Drugs.

[bb0140] Schuit S.C., van der Klift M., Weel A.E., de Laet C.E., Burger H., Seeman E., Hofman A., Uitterlinden A.G., van Leeuwen J.P., Pols H.A. (2004). Fracture incidence and association with bone mineral density in elderly men and women: the Rotterdam study. Bone.

[bb0145] Silverberg S.J., Shane E., Jacobs T.P., Siris E., Bilezikian J.P. (1999). A 10-year prospective study of primary hyperparathyroidism with or without parathyroid surgery. N. Engl. J. Med..

[bb0150] Stone K.L., Seeley D.G., Lui L.Y., Cauley J.A., Ensrud K., Browner W.S., Nevitt M.C., Cummings S.R. (2003). BMD at multiple sites and risk of fracture of multiple types: long-term results from the study of osteoporotic fractures. J. Bone Miner. Res..

[bb0155] Tadaka S., Saigusa D., Motoike I.N., Inoue J., Aoki Y., Shirota M., Koshiba S., Yamamoto M., Kinoshita K. (2018). jMorp: Japanese multi omics reference panel. Nucleic Acids Res..

[bb0160] Thakker R.V. (2016). Genetics of parathyroid tumours. J. Intern. Med..

[bb0165] Vargas-Poussou R., Huang C., Hulin P., Houillier P., Jeunemaître X., Paillard M., Planelles G., Déchaux M., Miller R.T., Antignac C. (2002). Functional characterization of a calcium-sensing receptor mutation in severe autosomal dominant hypocalcemia with a Bartter-like syndrome. J. Am. Soc. Nephrol..

[bb0170] Yamamoto M., Akatsu T., Nagase T., Ogata E. (1988). Comparison of hypercalcemic hypocalciuria (familial benign hypercalcemia) with primary hyperparathyroidism. Jpn. J. Med..

